# Right atrial appendage: an important structure to drive atrial fibrillation

**DOI:** 10.1007/s10840-021-01106-8

**Published:** 2022-02-18

**Authors:** Yang Liu, Ziliang Song, Weifeng Jiang, Shaohui Wu, Xu Liu, Mu Qin

**Affiliations:** Department of Cardiology, Shanghai Chest Hospital, Shanghai Jiao Tong University, 241 Huaihai West Road, Shanghai, China

**Keywords:** Atrial fibrillation, Right atrial appendage, Potential maps, Catheter ablation

## Abstract

**Purpose:**

Understanding of the atrial fibrillation (AF) driven by right atrial appendage (RAA) is limited. This study aimed to understand the characteristics of the AF driven by RAA and explore ablation methods.

**Methods:**

This was a retrospective study and patients who were identified as having the AF driven by RAA were reviewed. Ablation was performed during AF. Potential maps of the left and right atrium, electrophysiological examinations, and ablation methods were studied.

**Results:**

Among the 20 identified patients (mean age 67.0 ± 11.2 years; ejection fraction 62.9 ± 6.0%; LA diameter 43.1 ± 4.9 mm; RA diameter 51.7 ± 8.3 × 42.9 ± 3.7 mm), the AF cycle length in RAA (134.0 ± 10.9 ms) was the shortest, and the fastest frequency potentials were located in the RAA in 65% of patients. For the left atrium, the AF cycle length of the roof (145.5 ± 14.9 ms) was the shortest, followed by the left atrial appendage (153.7 ± 17.1 ms) and bottom (154.8 ± 11.8 ms). High-frequency potentials of RAA could be rapidly conducted to left atrium via sagittal bundle and Bachmann’s bundle, and the conduction time (55.0 ± 5.0 ms) was significantly shorter than the mean bi-atrial activation time (176.7 ± 10.3 ms, *P* < 0.0001). AF could be terminated after ablation at the RAA base (17 patients) or mechanical stimulation within the RAA (3 patients). To date, only two patients had recurrent atrial flutter, while the remaining patients maintained sinus rhythm.

**Conclusion:**

The AF driven by RAA is characterized by high-frequency potentials in RAA, and ablation at the RAA base can achieve a satisfactory therapeutic effect.

**Supplementary Information:**

The online version contains supplementary material available at 10.1007/s10840-021-01106-8.

## Introduction

In a previous study, the incidence of right atrial appendage (RAA) as a driver of atrial fibrillation (AF) was very low [1]. Subsequently, the special anatomy of RAA was focused by several studies and it was speculated to be related to the mechanism of arrhythmia. Recently, Ghannam et al. found that the RAA appeared to be a major source of fibrillation activity in the right atrium (RA) [2]. In addition, several basic studies also demonstrated that tissue heterogeneity of the pectinate structure in the RAA itself provided the conditions for the formation and maintenance of AF driver [3, 4]. However, there is still a lack of clinical evidences on the AF driven by RAA. We found that the proportion of AF mediated by RAA was not uncommon if routine bi-atrial mapping was performed during the procedure. The present study summarizes the characteristics of potentials and the ablation methods in the AF driven by RAA, and hopes to be able to help in the identification and treatment of this type of AF.

## Methods

### Study population

This was a retrospective and observational study at Shanghai Chest Hospital between March 2019 and April 2021, and patients with the AF driven by RAA were examined. Patients were included if their AF could be terminated (sinus rhythm or atrial flutter/atrial tachycardia) through intervening RAA during the procedure. Informed consent was not necessary due to the observational nature of the study. This study protocol was approved by the Institutional Ethics Committee at the Shanghai Chest Hospital.

### Mapping and catheter ablation

All patients were prepared preoperatively depending on the guidelines. A decapolar catheter was placed into the coronary sinus (CS), and the activated clotting time (ACT) was maintained between 300 and 350 s during the procedure. After two punctures of atrial septum, left atrium model was constructed with the use of a PentaRay catheter and the Carto 3 system (Biosense‐Webster). All patients underwent circumferential pulmonary vein isolation (CPVI) first during AF. The SMARTTOUCH Catheter or the THERMOCOOL SMARTTOUCH SF Catheter (Biosense Webster, Inc., Irvine, California) were used for ablation. Bi-atrial mapping was performed using a PentaRay catheter if AF was not terminated after CPVI. According to the previous literature, reference AF cycle length (AFCL) calculated manually by averaging 10 consecutive beats [5] was used to analyze the potentials in different sites of atria. The main part of the RAA was constructed using a PentaRay catheter in FAM (fast anatomical mapping) mode, and intracardiac echocardiography (ICE) was also utilized to further determine the ostium of the RAA. AF was identified to be possibly driven by RAA if high-frequency potentials were mapped inside or at the base of RAA. The high-frequency potentials were considered to be potentials with AFCLs equal to or shorter than left atrial potentials AFCLs. For the patients with similar frequencies of RAA and left atrial potentials, we initially ablated the left atrium in regions with relatively shorter AFCLs. If these procedures did not affect the condition of AF (termination or slowing of the AFCL), we performed the ablation of RAA. If the RAA potential had shorter AFCL compared with left atrial potentials, ablation of the RAA was performed, and no additional ablation of the left atrium was performed except for CPVI. For cases where the fastest frequency potentials were at the RAA base, ablation was performed in sheet form at the corresponding location of the base. For cases where the fastest frequency potentials were inside the RAA, linear ablation was performed on the lateral and septal sides of the base. The septal side was ablated to the superior vena cava (SVC) ostium, and the lateral side was ablated from the middle of the RAA vestibule to tricuspid valve (TV) after ICE identified the RAA ostium. If AF was terminated to atrial flutter (AFL)/atrial tachycardia (AT), the latter was mapped and ablated. Patients underwent transthoracic cardioversion if their AFL/AT could not be terminated after ablation.

### Electrophysiological study

To elucidate the pro-arrhythmic substrate of RAA, we used ICE and pacing mapping to study the anatomical structure and electrical conductivity characteristics in RAA under sinus rhythm. Multiple ultrasonic sectors of the RAA were obtained by moving the ultrasonic catheter in the right atrium. The contour of the RAA and the important structures including the sagittal bundle (SB) and terminal crest (TC) were constructed and shown in different colors under different ultrasound sectors. Then, the ablation catheter was placed at a specific location in the RAA and the pacing was initiated, while the local activation time (LAT) mapping was utilized to show the sequence of activation conduction in the right atrium. Finally, we inserted the PentaRay catheter into the left atrium and used the same method described above to explore the sequence of activation conduction from the RAA to the left atrium. The pacing current output was 10 mA.

### Follow-up

After ablation, the patients were hospitalized for at least 3 days and received continuous ECG monitoring during the first 48 h. At 1, 3, 6, 9, and 12 months after ablation, the patients went to the outpatient clinic for reexamination and received 24-h Holter monitoring. If follow-up information for a patient was not available in the hospital’s medical record system, we contacted the patient by phone. Patients were not routinely treated with antiarrhythmic drugs after ablation unless recurrence occurred. Recurrence was defined as sustained AF/AFL/AT after the 3-month blanking period.

### Statistical analysis

The data were presented as mean ± SD and median ± IQ for continuous variables, as appropriate. Continuous variables among different groups were compared by a one-way ANOVA test, and a *P* value < 0.05 was considered to be statistically significant. All analyses were performed using SPSS version 19.0 and GraphPad Prism version 8.2.1.

## Results

### Patient population

Twenty patients were included in the study from March 2019 to April 2021. Demographic, clinical, and echocardiographic characteristics of these patients are shown in Table 1. Mean age was 67.0 ± 11.2 years. Patients included in the study had a tendency to have a long history of AF, and the mean CHA2DS2-VASc score was 1.9 ± 1.2. In addition to enlargement of the left atria, which was often seen in patients with AF, patients in the study also appeared to have larger right atriums (51.7 ± 8.3 × 42.9 ± 3.7 mm).

**Table 1:** Characteristics of patients (*N* = 20)

Male	13 (65.0)
Age, years	67.0 ± 11.2
Hypertension	9 (45.0)
Diabetes mellitus	2 (10.0)
Coronary heart disease	2 (10.0)
CHA_2_DS_2_-VASc score	1.9 ± 1.2
Atrial fibrillation	
History of atrial fibrillation, months	31 (12, 90)
Paroxysmal atrial fibrillation	3 (15.0)
Persistent atrial fibrillation	9 (45.0)
Long-standing persistent atrial fibrillation	8 (40.0)
Left atrial diameter, mm	43.1 ± 4.9
Right atrial diameter, mm	51.7 ± 8.3 × 42.9 ± 3.7
Left ventricular ejection fraction, %	62.9 ± 6.0
Complications	0 (0)

Values are *N* (%), mean ± SD, or median (25th to 75th quartiles)

### Bi-atrial mapping

**Fig. 1:**
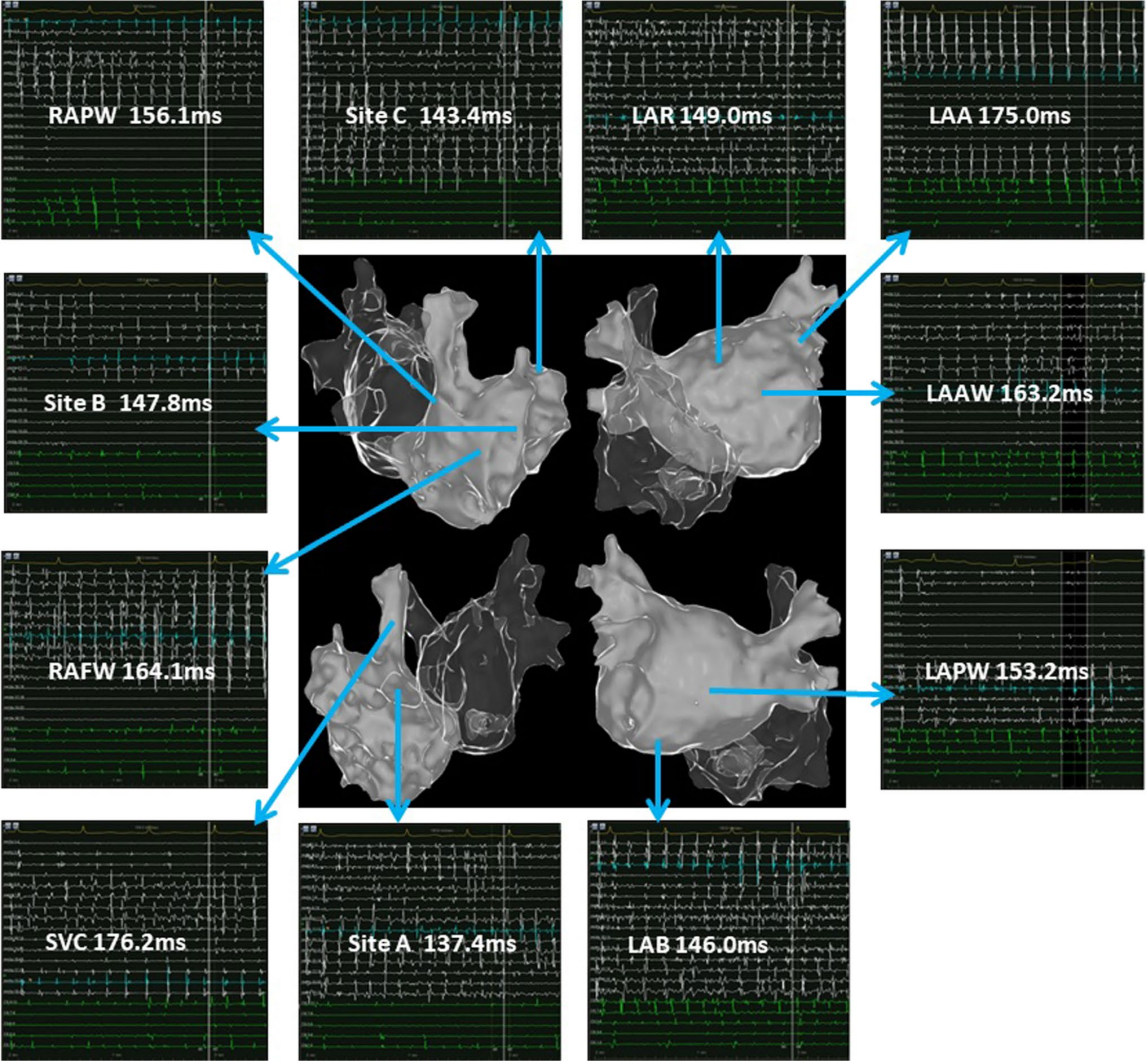
Potential maps of different regions of the left atrium and right atrium in patient 2. The potential frequency at the septal side of the right atrial appendage base was the fastest. For the left atrium, the potential frequency of the roof and bottom was relatively fast. In the potential maps, the white potential lines represent the bipolar potentials of the PentaRay electrodes and the green potential lines represent the bipolar potentials of the coronary sinus electrodes. Values represent the atrial fibrillation cycle lengths in the corresponding regions. LAR: left atrial roof; LAAW: left atrial anterior wall; LAPW: left atrial posterior wall; LAB: left atrial bottom; LAA: left atrial appendage; SVC: superior vena cava; RAPW: right atrial posterior wall; RAFW: right atrial free wall; site A: the septal side of the right atrial appendage base; site B: the free wall side of the right atrial appendage base; site C: inside of the right atrial appendage

**Fig. 2:**
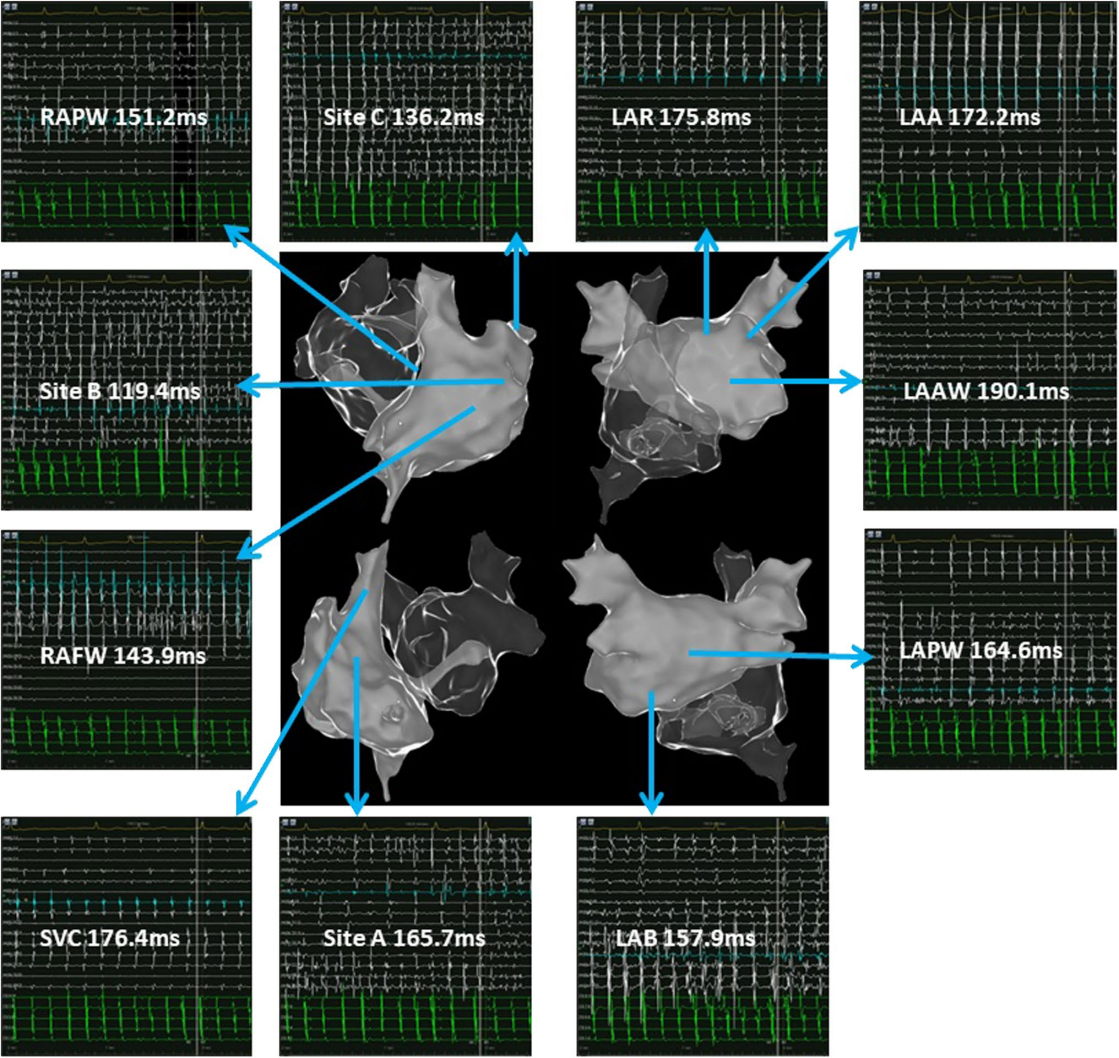
Potential maps of different regions of the left atrium and right atrium in patient 3. The potential frequency at the free wall side of the right atrial appendage base was the fastest. For the left atrium, the potential frequency of the bottom was the fastest. In the potential maps, the white potential lines represent the bipolar potentials of the PentaRay electrodes and the green potential lines represent the bipolar potentials of the coronary sinus electrodes. Values represent the atrial fibrillation cycle lengths in the corresponding regions. LAR: left atrial roof; LAAW: left atrial anterior wall; LAPW: left atrial posterior wall; LAB: left atrial bottom; LAA: left atrial appendage; SVC: superior vena cava; RAPW: right atrial posterior wall; RAFW: right atrial free wall; site A: the septal side of the right atrial appendage base; site B: the free wall side of the right atrial appendage base; site C: inside of the right atrial appendage

**Fig. 3:**
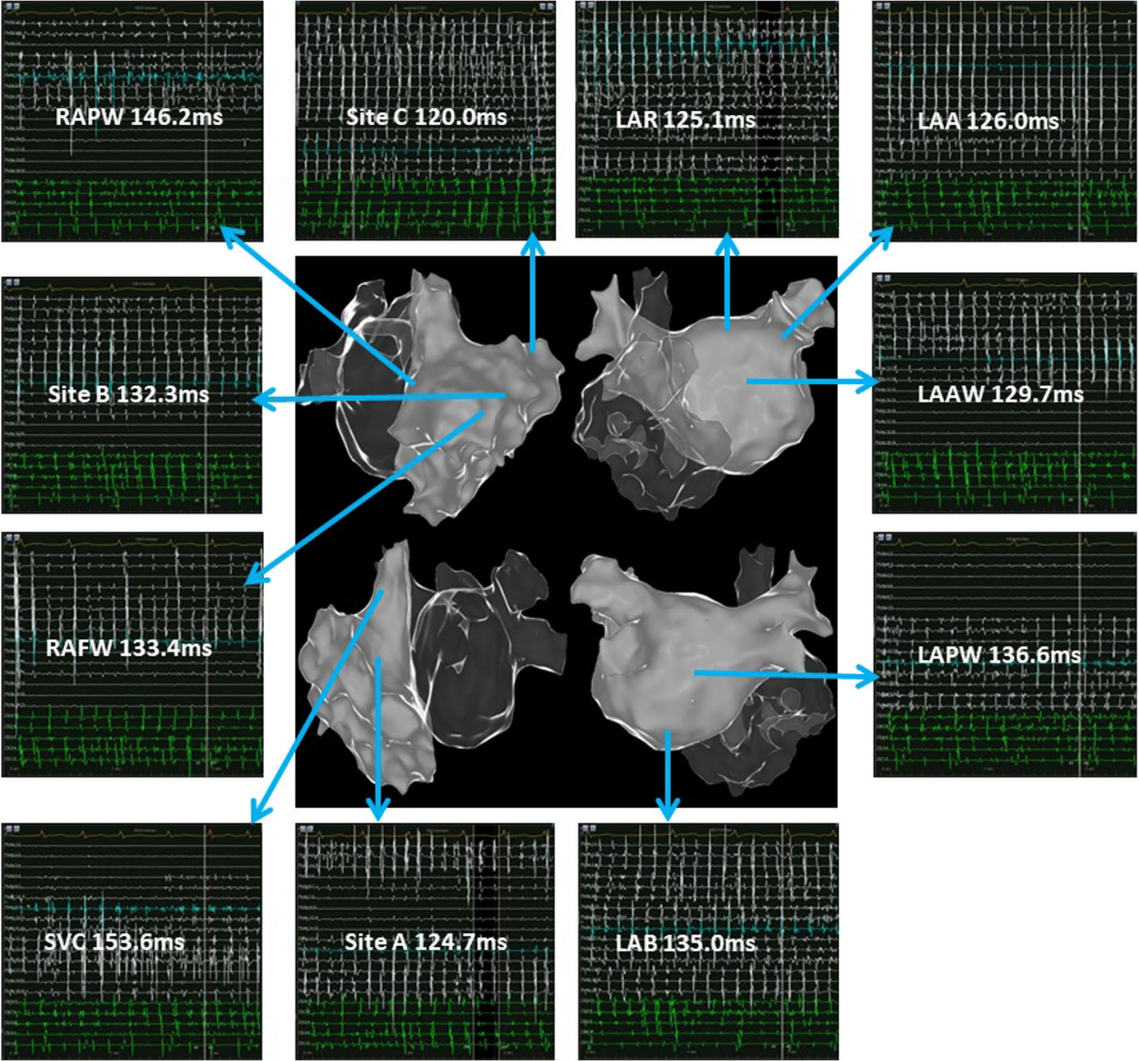
Potential maps of different regions of the left atrium and right atrium in patient 12. The potential frequency inside the right atrial appendage was the fastest. For the left atrium, there were obvious high-frequency potentials at the roof, bottom, and left atrial appendage. In the potential maps, the white potential lines represent the bipolar potentials of the PentaRay electrodes and the green potential lines represent the bipolar potentials of the coronary sinus electrodes. Values represent the atrial fibrillation cycle lengths in the corresponding regions. LAR: left atrial roof; LAAW: left atrial anterior wall; LAPW: left atrial posterior wall; LAB: left atrial bottom; LAA: left atrial appendage; SVC: superior vena cava; RAPW: right atrial posterior wall; RAFW: right atrial free wall; site A: the septal side of the right atrial appendage base; site B: the free wall side of the right atrial appendage base; site C: inside of the right atrial appendage

**Fig. 4:**
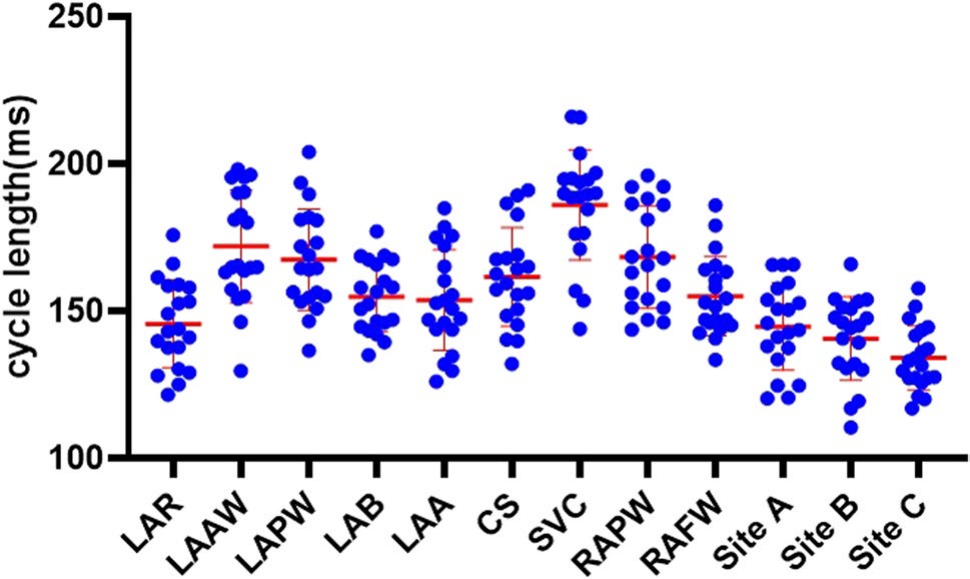
Scatter diagram of the atrial fibrillation cycle lengths in different regions of the left and right atrium. LAR: left atrial roof; LAAW: left atrial anterior wall; LAPW: left atrial posterior wall; LAB: left atrial bottom; LAA: left atrial appendage; CS: coronary sinus; SVC: superior vena cava; RAPW: right atrial posterior wall; RAFW: right atrial free wall; site A: the septal side of the right atrial appendage base; site B: the free wall side of the right atrial appendage base; site C: inside of the right atrial appendage

Average sampling points of bi-atrial high precision mapping were 1880 ± 445, and the mean mapping time was 16.9 ± 4.0 min. Based on the specificity of RAA and its surrounding anatomical structures, site A, site B, and site C were used to represent the septal side of the RAA base, the free wall side of the RAA base, and the inside of the RAA, respectively. AF cycle lengths in different segments of the left atrium and right atrium and their representative potential maps are shown in Table 2, Fig. 1, Fig. 2, and Fig. 3. The area with the shortest local average AFCL was RAA during bi-atrial mapping (134.0 ± 10.9 ms). The roof as the area with the fastest potential frequency in the left atrium, its local AFCL (145.5 ± 14.9 ms) is slightly longer than that of RAA. For the left atrium, potential frequencies of the LAA (153.7 ± 17.1 ms) and bottom (154.8 ± 11.8 ms) were also relatively fast, although slightly slower than that of the roof. The above results are presented in Fig. 4. In the present study, the fastest frequency potentials were located within the RAA in 65% of the enrolled patients, and the proportions of the fastest frequency potentials at the septal side and free wall side of the RAA base were 25% and 10%, respectively (Supplemental Fig. 1).

**Table 2:** Atrial fibrillation cycle lengths (ms) in different regions

Patient	LAR	LAAW	LAPW	LAB	LAA	CS	SVC	RAPW	RAFW	Site A	Site B	Site C
Patient 1	158.5	163.7	204.1	156.6	150.8	157.3	171.1	163.1	165.4	124.7	150.8	127.3
Patient 2	149.0	163.2	153.2	146.0	175.0	148.4	176.2	156.1	164.1	137.4	147.8	143.4
Patient 3	175.8	190.1	164.6	157.9	172.2	165.1	176.4	151.2	143.9	165.7	119.4	136.2
Patient 4	152.6	164.9	173.2	160.1	143.8	182.8	216.0	196.1	145.2	120.5	139.3	125.7
Patient 5	153.2	198.2	171.9	167.6	153.6	164.3	189.5	167.9	140.7	159.6	132.0	127.6
Patient 6	139.8	164.8	164.1	150.7	147.1	159.4	215.8	192.2	154.3	150.7	153.9	141.7
Patient 7	128.0	180.0	155.1	158.0	146.0	156.1	193.8	168.4	160.6	153.8	147.6	147.5
Patient 8	121.6	154.2	156.3	139.4	134.7	140.3	143.9	147.1	142.5	143.6	140.7	127.3
Patient 9	143.0	164.7	156.4	152.4	143.7	162.6	188.8	154.1	147.1	145.9	145.1	116.9
Patient 10	166.0	182.7	193.5	168.7	165.2	186.5	194.5	181.1	152.9	165.7	130.5	134.0
Patient 11	161.4	195.4	189.7	165.8	152.7	189.3	196.9	192.3	179.1	133.6	110.4	131.5
Patient 12	125.1	129.7	136.6	135.0	126.0	145.3	153.6	146.2	133.4	124.7	132.3	120.0
Patient 13	129.0	196.3	146.5	177.0	175.6	191.0	203.6	186.4	163.3	141.2	151.2	144.5
Patient 14	143.9	165.4	164.5	147.0	155.4	155.6	184.5	151.1	146.0	142.5	116.9	151.5
Patient 15	157.9	190.4	180.8	167.4	178.5	167.9	188.5	188.2	185.9	165.8	165.9	157.7
Patient 16	141.0	181.0	181.8	143.3	184.9	150.6	195.1	165.5	151.5	150.4	144.3	137.2
Patient 17	158.9	195.4	181.1	168.8	160.3	167.5	189.9	186.1	158.0	157.7	146.1	133.0
Patient 18	137.6	146.3	150.8	142.0	131.9	139.8	156.8	143.7	147.0	138.1	130.1	120.9
Patient 19	137.7	157.3	168.9	146.6	147.4	169.0	190.0	170.6	171.5	152.7	153.3	129.7
Patient 20	130.3	155.0	154.6	144.7	129.7	132.0	194.8	158.8	147.0	120.3	154.1	127.2
Mean ± SD	145.5 ± 14.9	171.9 ± 19.1	167.4 ± 17.2	154.8 ± 11.8	153.7 ± 17.1	161.5 ± 16.7	186.0 ± 18.7	168.3 ± 17.4	155.0 ± 13.5	144.7 ± 14.8	140.6 ± 14.2	134.0 ± 10.9

LAR: left atrial roof; LAAW: left atrial anterior wall; LAPW: left atrial posterior wall; LAB: left atrial bottom; LAA: left atrial appendage; CS: coronary sinus; SVC: superior vena cava; RAPW: right atrial posterior wall; RAFW: right atrial free wall; site A: the septal side of the right atrial appendage base; site B: the free wall side of the right atrial appendage base; site C: inside of the right atrial appendage

### Ablation outcomes

Complete CPVI was achieved in all included patients. When using a PentaRay catheter to map the RAA, AF was terminated to sinus rhythm in three patients due to the mechanical stimulation of the catheter. These three patients had paroxysmal AF. Another 17 patients underwent RAA ablation, all at the RAA base, and the mean ablation time for RAA was 14.6 ± 8.1 min. According to the results of the mapping, the fastest frequency potentials were located at the free wall side of the RAA base in two patients, and AF was terminated after ablation in local patches, with one patient converting to sinus rhythm and the other to AFL. The mean ablation area of the RAA base was 318.0 ± 25.5 mm^2^, and the mean ablation time for RAA was 19.0 ± 1.4 min. AF was terminated in five patients after sheet ablation only at the septal side of the RAA base, with two patients converting to sinus rhythm and the others to AFL. The mean ablation area of the RAA base was 86.4 ± 19.7 mm^2^, and the mean ablation time for RAA was 6.0 ± 1.6 min. Mapping results of the other 10 patients showed that the high-frequency potentials were located in the RAA, so linear ablation was applied to the base of the RAA (both free wall side and septal side). The mean ablation area of the RAA base was 301.2 ± 136.0 mm^2^, and the mean ablation time for RAA was 18.1 ± 7.6 min. AF was terminated after ablation with one patient converting to sinus rhythm and the others to AFL (Video). None of the patients had complications such as pericardial tamponade or thromboembolism. Representative ablation maps are shown in Supplemental Fig. 2.

### The electro-anatomical relationship between RAA and atrium

**Fig. 5:**
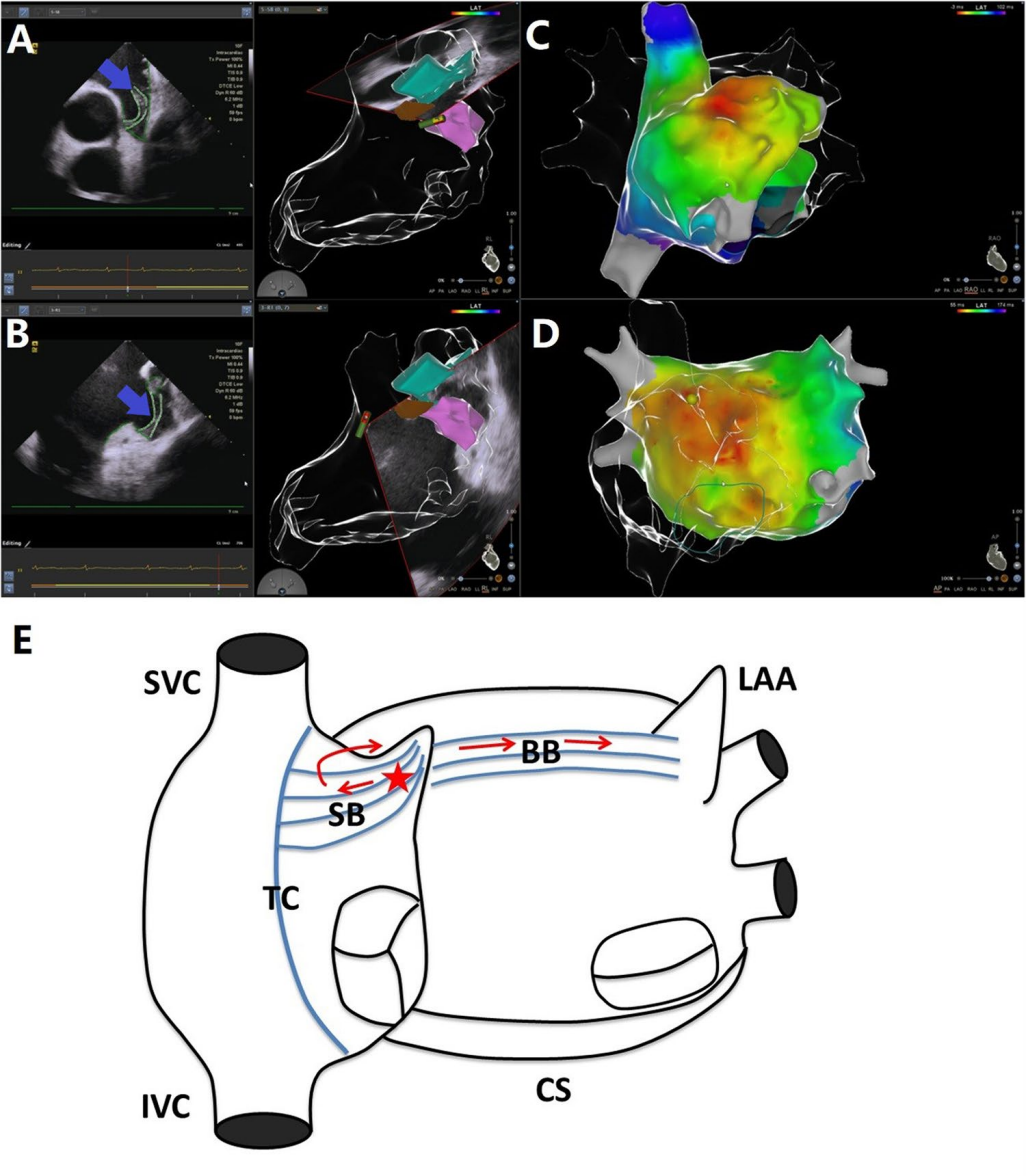
**A, B** Structures in the right atrial appendage under intracardiac echocardiography. The blue arrows indicate the sagittal bundle.  : terminal crest;  and  : sagittal bundle. **C**, **D** Results of local activation time mapping when paced at the yellow spot in the right atrial appendage. For this patient, the conduction time from SB pacing to the earliest point of left atrium activation was 55 ms, and the bi-atrial activation time was 174 ms. The corresponding data of the other two patients who underwent local activation time mapping were 60 ms, 188 ms and 50 ms, 168 ms, respectively. **E** The activation in the right atrial appendage (marked by the red star) was transmitted from sagittal bundle to terminal crest, to right atrial septum and finally to left atrium by Bachmann’s bundle. SB: sagittal bundle; TC: terminal crest; BB: Bachmann’s bundle; SVC: superior vena cava; IVC: inferior vena cava; LAA: left atrial appendage; CS: coronary sinus

According to the observation of the structure within RAA by ICE, we found that there were thick SBs along the long axis of RAA, which were formed by the convergence of multiple muscle bundles from TC. They went distally to the RAA and gave off branches reflexed to connect with the vestibule between the tricuspid annulus and the RAA, forming a circular muscular structure (Fig. 5A, B). In three patients with paroxysmal AF, we paced at the yellow spot which was located at the distal of SB after they transitioned to sinus rhythm, and the LAT mapping showed that the activation was transmitted from SB to TC, to right atrial septum, and finally to left atrium by Bachmann’s bundle (BB) (Fig. 5C–E). The mean LAT from SB pacing to the earliest point of left atrium activation was 55.0 ± 5.0 ms, which was significantly shorter than the mean bi-atrial activation time (176.7 ± 10.3 ms, *P* < 0.0001). This excitation feature suggested a dominant conduction pathway among the RAA, TC, and BB, which was consistent with the trend of bipolar potential frequency measured in AF.

### Follow-up

Because of the different dates of ablation, not all patients completed the four post-ablation follow-ups. All patients completed 1-, 3-, and 6-month follow-up after ablation, and the number of patients who completed 9- and 12-month follow-up after ablation was 16 and 13. No patients have had recurrent AF so far, which was a satisfactory result. In terms of maintaining sinus rhythm, one patient had AFL and 95.0% (19/20) of including patients maintained sinus rhythm at 1 month and 3 months after ablation. Six months after ablation, one patient who maintained sinus rhythm during the previous two follow-ups had AFL, and 90.0% (18/20) of patients maintained sinus rhythm. Sinus rhythm was maintained in 87.5% and 84.6% of patients at 9 and 12 months after ablation, respectively. The above results are shown in Supplemental Fig. 3.

## Discussion

The RAA is an important substrate for the maintenance of AF, and its role is often neglected. This study found the relationship between the atrial potential characteristics and anatomical characteristics of the AF driven by RAA. The SB-TC-BB dominant conduction pathway which could transmit high-frequency potentials from the RAA to the left atrium was the structural basis of this characteristic. The termination of AF could be achieved through ablation and improvement of this substrate.

The RAA forms the anterior wall of the atrium and is composed of ridges formed by pectinate muscles, which arise from the prominent crista terminalis [6]. A previous study which was carried out on the basis of echocardiography imaging reported that the area of RAA was bigger in patients with AF than in patients with sinus rhythm [7]. Besides, other studies indicated that RAA fibrosis and varied shape had an impact on the occurrence and maintenance of AF [8–11]. In our cases, the right atriums were enlarged to varying degrees, and the special anatomical structure in the RAA was also the key factor for the formation of AF driver. A study on optical mapping of human RAA showed that architectural discontinuity between pectinate muscles and small intramural bundles could lead to longitudinal and intramural conduction blocks and increased fibrosis could worsen the architectural discontinuity, which ultimately could create a structural substrate for AF driver [12]. This suggested that the RAA had anatomic substrates for AF formation.

The involvement of the right atrium in the maintenance of AF has been found in previous clinical studies [2, 13], and similar findings had been reported in our previous study [14]. However, the proportion of right atrial driver was low. This may be related to the non-routine bi-atrial mapping and the neglect of the role of RAA. In our study, we found that in the AF driven by RAA, the left atrial local potential also presented a certain degree of high-frequency potentials, especially at the BB insertion point of the roof and within LAA. This phenomenon may misjudge the results of the mapping and result in left atrial excessive ablation. In 13 (65%) cases in our study, the true driver of AF was found to be located in RAA after the corresponding region of the left atrium was ablated first. The mechanism of this potential characteristic was due to the existence of SB-TC-BB dominant conduction pathway. It has been shown that the conduction between the atria occurs mainly in BB [15]. The superior arm of the rightward extension of BB arises in the vicinity of the SB, and the inferior arm arises in the subepicardium of the right atrial vestibule [16]. The left side of BB is inserted into the anterior top of the left atrium and extends to the LAA [17]. In our study, through pacing RAA, it was found that the conduction time of SB to the position of the junction between BB and left atrium was significantly shorter than the activation time of other parts of the atrium, indicating that driver of RAA could rapidly spread to the left atrium through this pathway. This is also why the potential frequencies in other parts of the right atrium were slower than that of the left atrial roof. Notably, interatrial bundles are not limited to the BB and are present in all areas of the interatrial septum. Besides, inferiorly located bundles can sometimes be more prominent than the BB [18]. This explains why the frequency of the left atrial bottom potential was very fast in some patients.

Our strategy for ablation of RAA is as follows: (1) For AF driven by the base of RAA, ablation was performed in sheet form at the corresponding septal or free wall side of the RAA base. (2) For cases where the driver was located in the RAA, the dominant SB-BB pathway was blocked by linear ablation at the septal and free wall side of the RAA base. Immediate termination of AF was achieved by this ablation strategy, although the driver in the RAA was not directly interfered with and this linear ablation was difficult to achieve complete blockade of conduction. We speculated that it may be due to the substrate improvement effect. In a previous study [13], ablation in the RAA was guided by the Lasso catheter, which was inserted into the body of the RAA, with ablation proximal to the catheter, and this method was reported to be able to terminate AF. The ablation method recently reported by Ghannam et al. was circumferential ablation around the RAA [2]. Because of the fact that RAA has pectinate, smooth-walled vestibule and low blood flow [19], catheter ablation within RAA is complicated. In addition, the shallow nature of the atrial appendage increases the likelihood that current catheter manipulation within RAA may easily lead to cardiac perforation. Thus, compared with the above two methods, our ablation method is safer and reduces ablation damage while terminating AF. Certainly, we look forward to better intervention techniques for the atrial appendage in the future.

### Limitations

First, the number of cases contained in the present study was relatively small, and we did not set a propensity score matched group, which in our opinion would not be significant for such a small sample. Since the main purpose of this study was to summarize the characteristics and ablation methods of the AF driven by RAA, a control group was not very necessary. Second, the relationship between the morphology of RAA and AF has not been carefully studied due to the absence of some relevant materials. Last but not least, further studies are needed to confirm the efficacy of this ablation approach and to determine the detailed mechanism and role of RAA ablation in AF.

## Conclusions

The left atrial potential characteristics of the AF driven by RAA, which was related to the SB-TC-BB dominant conduction pathway between the RAA and left atrium, may easily lead to the misjudgment of drivers. For this type of AF, ablation at the RAA base could lead to the termination of AF in a safe and long-term manner.

## Supplementary Information

Below is the link to the electronic supplementary material.

Supplementary file1 (PDF 568 KB)

During the linear ablation at the right atrial appendage base, atrial fibrillation was terminated to atrial flutter (MP4 342 KB)
