# Supplementary material for: Right atrial appendage: an important structure to drive atrial fibrillation
Source: J Interv Card Electrophysiol. 2022 Feb 18;65(1):73–82. doi: 10.1007/s10840-021-01106-8 (PMC9550756; doi:10.1007/s10840-021-01106-8)
Supplement: Supplementary file 1 — Supplementary file1 (PDF 568 KB) [file 10840_2021_1106_MOESM1_ESM.pdf]

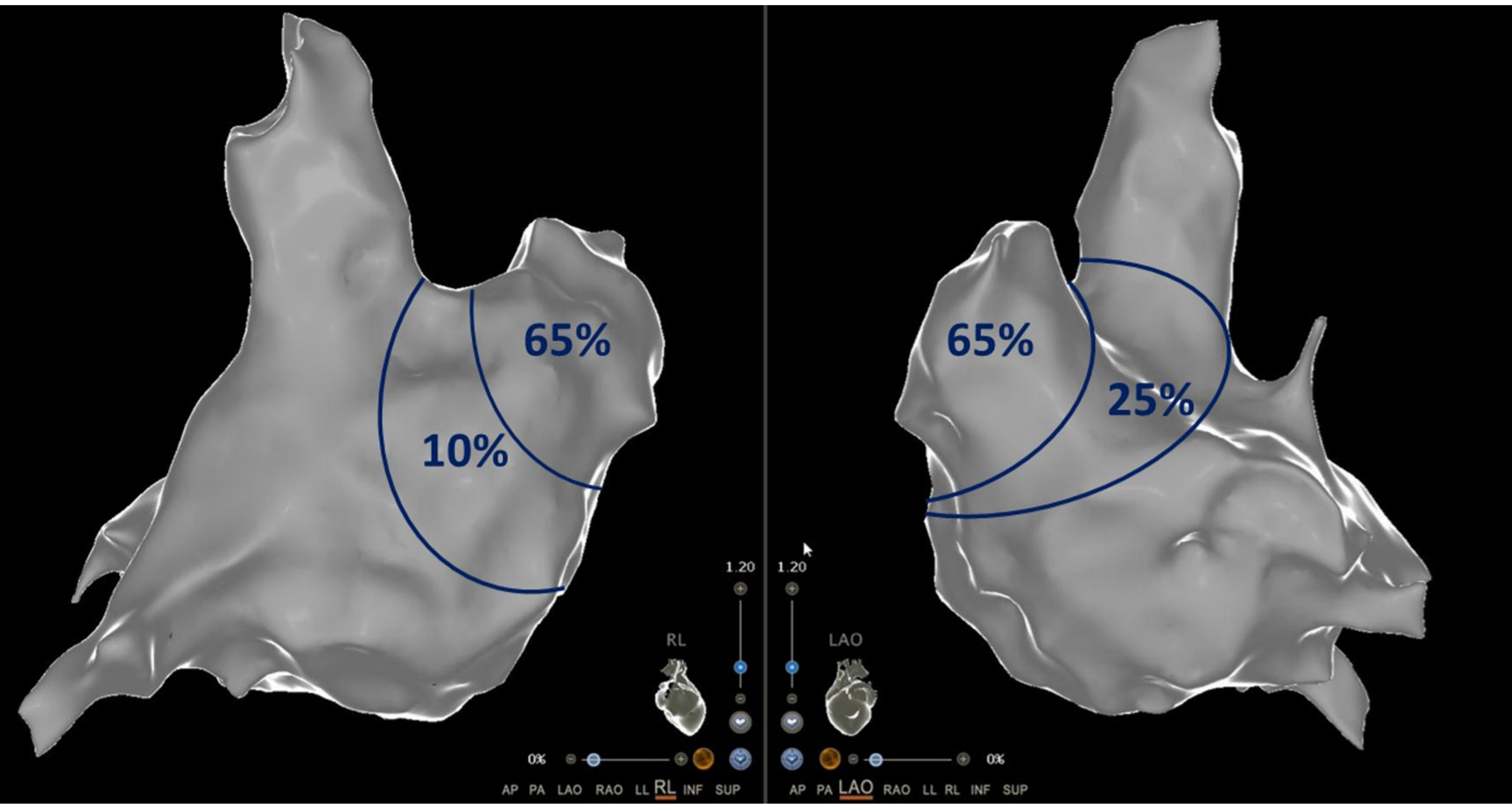

Supplemental Figure 1. Distribution of high frequency potentials in the right atrial appendage. The fastest frequency potentials were located within the RAA in 65% of the enrolled patients, and the proportions of the fastest frequency potentials at the septal side and free wall side of the RAA base were 25% and 10%, respectively.

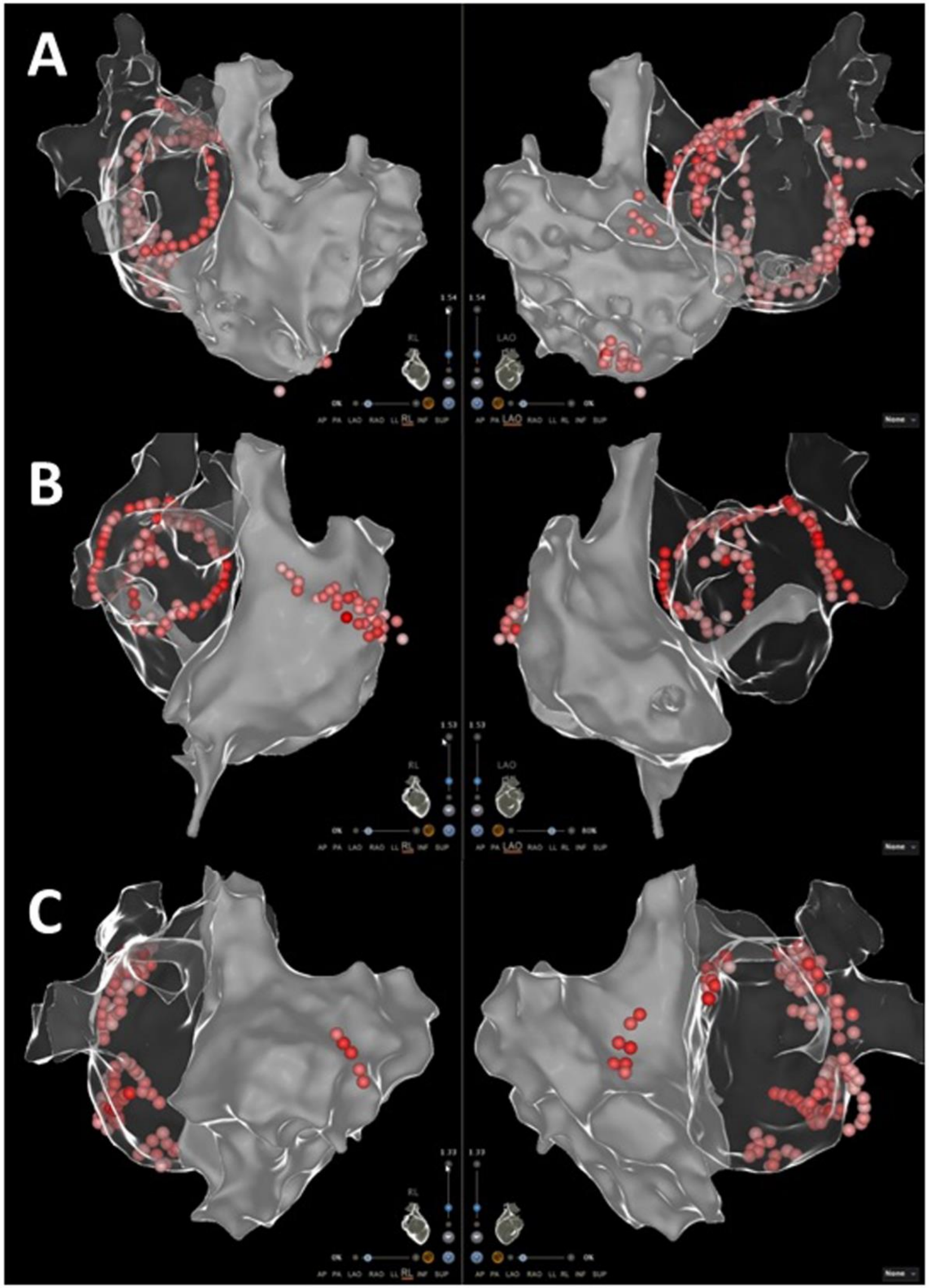

Supplemental Figure 2. Ablation results. A: ablation was performed in sheet form at the septal side of the right atrial appendage base. B: ablation was performed in sheet form at the free wall side of the right atrial appendage base. C: linear ablation was performed at both the septal and free wall sides of the right atrial appendage base.

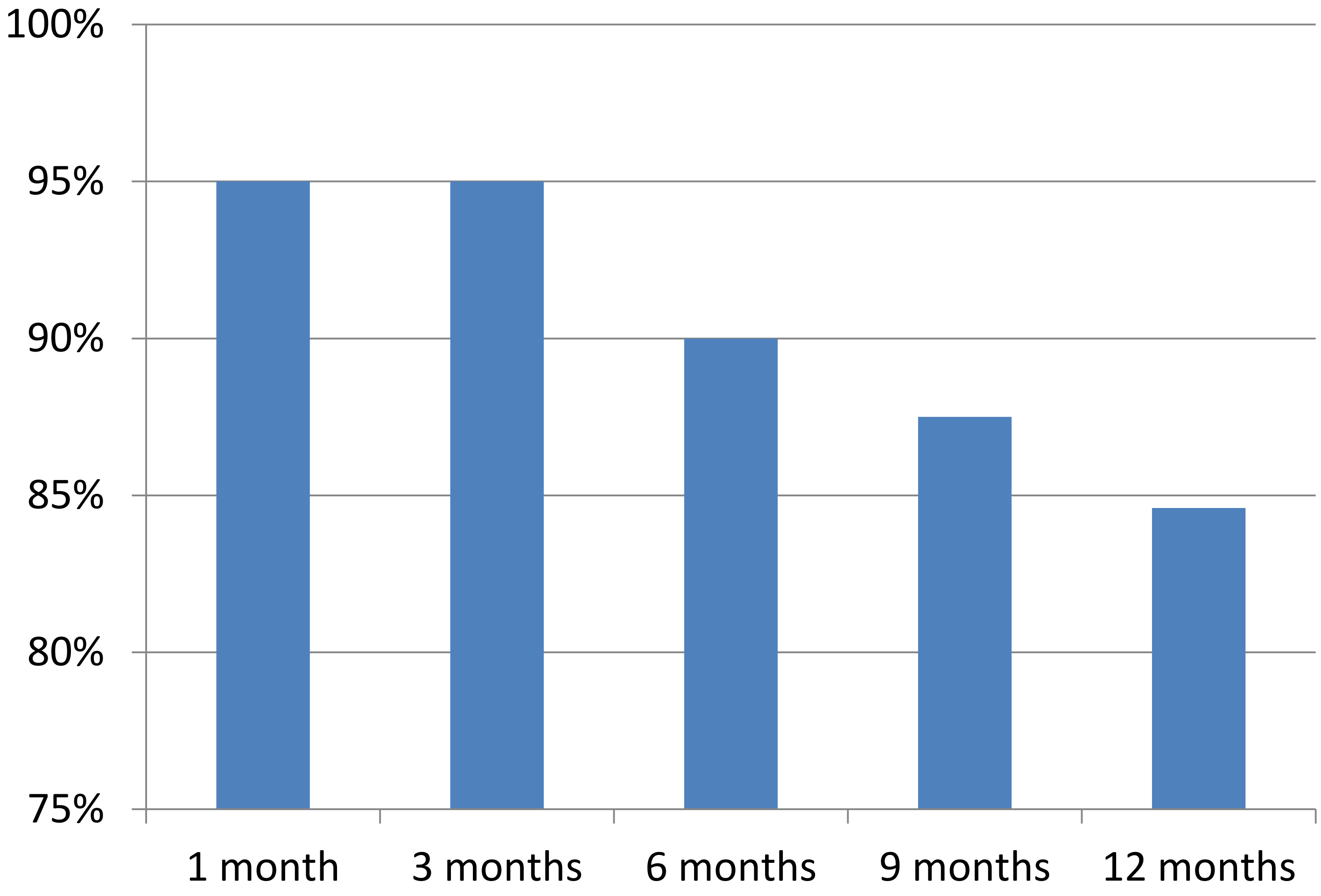

Supplemental Figure 3. Proportion of patients maintaining sinus rhythm after ablation. All patients completed 1-, 3- and 6-month follow-up after ablation, and the number of patients who completed 9- and 12-month follow-up after ablation was 16 and 13.
